# Differential Methylation and Acetylation as the Epigenetic Basis of Resveratrol’s Anticancer Activity

**DOI:** 10.3390/medicines6010024

**Published:** 2019-02-13

**Authors:** Mohd Farhan, Mohammad Fahad Ullah, Mohd Faisal, Ammad Ahmad Farooqi, Uteuliyev Yerzhan Sabitaliyevich, Bernhard Biersack, Aamir Ahmad

**Affiliations:** 1 College of Basic Sciences, King Faisal University, Hofuf 400-Al Ahsa-31982, Saudi Arabia; mfarhan@kfu.edu.sa; 2 Department of Medical Laboratory Technology, Faculty of Applied Medical Sciences, University of Tabuk, P.O. Box 741, Tabuk 71491, Saudi Arabia; m.ullah@ut.edu.sa; 3 Department of Psychiatry, University Hospital Limerick, Limerick V94 T9PX, Ireland; mohd.faisal@hse.ie; 4 Institute of Biomedical and Genetic Engineering (IBGE), Islamabad 44000, Pakistan; ammadfarooqi@rlmclahore.com; 5 Department of Postgraduate Education and Research, Kazakhstan Medical University KSPH, Almaty 050004, Kazakhstan; e.uteuliyev@ksph.kz; 6 Organic Chemistry Laboratory, Department of Chemistry, University of Bayreuth, Universitaetsstrasse 30, 95447 Bayreuth, Germany; bernhard.biersack@yahoo.com; 7 Department of Oncologic Sciences, Mitchell Cancer Institute, University of South Alabama, Mobile, AL 36604, USA

**Keywords:** resveratrol, epigenetic, methylation, acetylation

## Abstract

Numerous studies support the potent anticancer activity of resveratrol and its regulation of key oncogenic signaling pathways. Additionally, the activation of sirtuin 1, a deacetylase, by resveratrol has been known for many years, making resveratrol perhaps one of the earliest nutraceuticals with associated epigenetic activity. Such epigenetic regulation by resveratrol, and the mechanism thereof, has attracted much attention in the past decade. Focusing on methylation and acetylation, the two classical epigenetic regulations, we showcase the potential of resveratrol as an effective anticancer agent by virtue of its ability to induce differential epigenetic changes. We discuss the de-repression of tumor suppressors such as BRCA-1, nuclear factor erythroid 2-related factor 2 (NRF2) and Ras Associated Domain family-1α (RASSF-1α) by methylation, PAX1 by acetylation and the phosphatase and tensin homologue (PTEN) by both methylation and acetylation, in addition to the epigenetic regulation of oncogenic NF-κB and STAT3 signaling by resveratrol. Further, we evaluate the literature supporting the potentiation of HDAC inhibitors and the inhibition of DNMTs by resveratrol in different human cancers. This discussion underlines a robust epigenetic activity of resveratrol that warrants further evaluation, particularly in clinical settings.

## 1. Introduction

**Figure 1:**
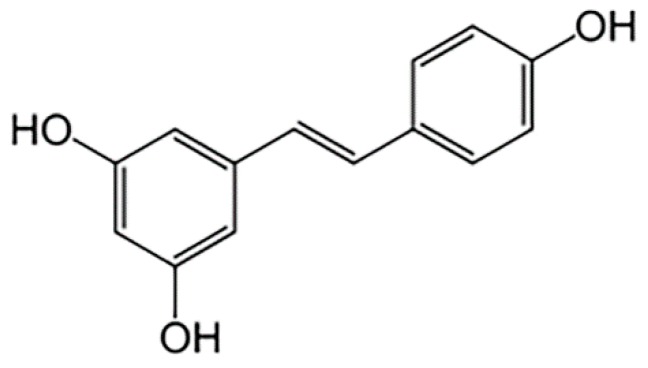
Chemical structure of resveratrol.

Resveratrol (3,5,4′-trihydroxy-*trans*-stilbene) (Figure 1) is a naturally occurring polyphenol found in peanuts and the skin of grapes and berries. It is a phytoalexin produced in response to injury, ultraviolet radiation or a pathogen attack. The initial interest in resveratrol was because of its antioxidant properties [1], which led to the recognition of its chemopreventive ability [2]. Resveratrol was also found to generate reactive oxygen species, leading to effective anticancer activity through a prooxidant mechanism [3,4] which also happens to be a hallmark of several other polyphenols [5,6,7]. It is now believed that resveratrol exhibits both antioxidant and prooxidant properties [8,9,10], which depend largely on the tumor microenvironment and the presence of transition metal ions, particularly copper ions. Besides its anticancer properties, resveratrol has also been investigated to explain the ‘French paradox’ [11], a phenomenon of the relatively low incidence of coronary heart diseases in the French population despite a diet rich in saturated fats, perhaps due to the consumption of red wine with high resveratrol content. 

The realization of the potent anticancer properties of resveratrol was followed by numerous investigations into the various signaling pathways that it affects, leading to the observed effects [12,13,14,15,16,17]. In recent years, the focus of cancer research has shifted to stem cells, epithelial-to-mesenchymal transition (EMT) and epigenetic regulation, even in the context of lead compounds such as resveratrol that have a natural origin. Accordingly, a number of studies have documented the ability of resveratrol to affect stem cell populations [18,19] and EMT [20,21]. This article focuses on epigenetic regulation by resveratrol, which is increasingly being proposed as contributing to its anticancer properties.

## 2. Epigenetic Regulation by Resveratrol

Even though ‘epigenetics’ was originally intended to define heritable changes in phenotypes that were independent of changes in the DNA sequence, the meaning of this word has now considerably broadened to describe the alterations in human chromatin that influence DNA-templated processes [22,23]. Epigenetic changes do not result in any changes in the DNA sequence, but can still have lasting effects on gene expression. Epigenetic changes involve at least four known modifications of DNA and sixteen classes of histone modifications [22,23,24]. 

A number of studies have recognized the effects of nutraceuticals, including polyphenol resveratrol, on the epigenetic machinery in humans [25,26,27,28,29,30,31,32,33,34,35]. Resveratrol can modulate epigenetic patterns by altering the levels of S-adenosylmethionine and S-adenosylhomocysteine or by directing the enzymes that catalyze DNA methylation and histone modifications [27]. It also activates the deacetylase sirtuin and regulates oncogenic and tumor suppressor micro-RNAs [36]. Methylation and acetylation are two classical epigenetic modifications, and these will be the subject of our discussion in this article as we showcase the epigenetic basis of the anticancer action of resveratrol.

## 3. Methylation

The DNA methylation of gene promoters has a profound effect on their eventual expression and function. Differential DNA methylation can lead to a disease condition in healthy phenotypes. This is particularly true for cancer wherein the differential DNA methylation of the promoters of oncogenes and tumor suppressor genes helps maintain a delicate balance. The increased (hyper) methylation of promoter CpG islands causes gene silencing, while the decreased (hypo) methylation of promoter CpG islands leads to the expression of the gene. 

### 3.1. Breast Cancer

Breast cancer is a widely studied cancer when it comes to epigenetic regulation, particularly differential methylation, by resveratrol. Studies have been conducted to elucidate the genome-wide methylation patterns after resveratrol treatment, and there have also been efforts to understand the epigenetic basis of the de-repression of tumor suppressors by resveratrol. The subsection to follow will focus on this activity of resveratrol.

#### 3.1.1. Genome-Wide Analyses

A study on the genome-wide methylation-modifying effects of resveratrol, which involved exposing MCF10CA1h and MCF10CA1a cells to resveratrol for 9 days followed by Illumina 450K array analysis, revealed a profound effect of resveratrol on genome-wide DNA methylation with approximately 75% differentially methylated genes being hypermethylated [37]. This study evaluated the epigenetic activity of not only resveratrol but also pterostilbene, an analog of resveratrol. While treatment with resveratrol was done at a dose of 15 μM, pterostilbene treatment was at a 7 μM dose. The compounds targeted genes that are over-expressed in tumors because of DNA hypomethylation. Since increased DNA methylation results in the silencing of genes, it makes sense that the resveratrol-induced hypermethylated genes have predominantly oncogenic functions [37], such as the Notch signaling pathway. However, in another study that also looked at genome-wide DNA methylation after resveratrol treatment (in this case, 24 h and 48 h treatment), only about 12.5% of CpG loci were found to be differentially methylated by resveratrol [38]. This study used MDA-MB-231 breast cancer cells and the concentration of resveratrol was 100 μM. Additionally, this study predominantly found DNA hypomethylation by resveratrol. It is possible that the differences in genome-wide DNA methylation (75% vs. 12.5%) in these two studies might be reflective of the different experimental setups, in particular, the time-durations of the resveratrol treatment as well as the doses used, with longer treatment leading to substantially more methylation.

#### 3.1.2. Effects on Tumor Suppressors

**Figure 2:**
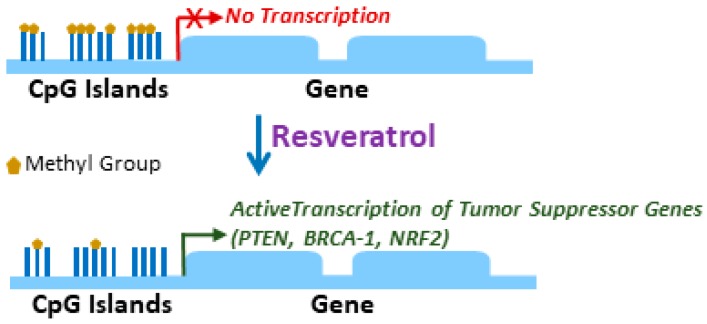
Activation of tumor suppressor genes by resveratrol through promoter DNA hypomethylation. The CpG islands in the DNA promoter regions of the tumor suppressor genes are hypermethylated resulting in their silencing. De-methylation of these CpG islands by resveratrol results in the activation of transcription and the eventual expression of tumor suppressor genes such as the phosphatase and tensin homologue (PTEN), BRCA-1 and nuclear factor erythroid 2-related factor 2 (NRF2).

Tumor suppressor genes are typically inactivated at the onset of tumorigenesis and, therefore, their reactivation by anticancer therapy is a mechanism through which tumor progression can be controlled. Several studies have documented the epigenetic reactivation of the tumor suppressor genes by resveratrol in breast cancer (Figure 2), as discussed below.

Phosphatase and tensin homologue (PTEN) is a well-characterized tumor suppressor in breast cancer [39]. In one of the earlier studies describing an effect of resveratrol on promoter DNA methylation, it was reported that resveratrol was highly efficient in reducing PTEN promoter DNA methylation in MCF7 breast cancer cells [40]. Since reduced DNA methylation leads to gene expression, this action of resveratrol induced the expression of PTEN which could explain its anticancer effects. Further, it was reported that the epigenetic effects were complex as not only PTEN was induced but the cell cycle regulator p21 was up-regulated as well, in addition to the down-regulation of DNMT (DNA methyltransferase). This is interesting because DNA methyltransferases increase methylation. Thus, it appears that resveratrol is able to reduce DNA methylation possibly through two different ways—by reducing methylation and by inducing de-methylation.

BRCA-1 (breast cancer type 1) is another tumor suppressor gene. Its expression is known to be regulated by epigenetic mechanisms. In a study that demonstrated the effects of resveratrol on BRCA-1 methylation [41], it was shown that exposure of breast cancer cells MCF7 to tumor promoter TCDD (2,3,7,8 tetrachlorodibenzo-p-dioxin) resulted in the hypermethylation of BRCA-1 promoter CpG island concomitant with an increased association of trimethylated histone H3K9 and DNMTs, namely DNMT1, DNMT3a and DNMT3b, with BRCA-1 promoter. Resveratrol, at physiologically relevant doses, was able to repress these TCDD effects. In a follow-up study, the research group evaluated the effects of TCDD alone, and in combination with resveratrol, on pregnant Sprague–Dawley rats [42]. It was found that, similar to in vitro observations in MCF7 cells, gestational exposure to TCDD led to the reduced DNA CpG island methylation of BRCA-1 promoter in the mammary tissues of the offspring, which was preceded by the occupation of BRCA-1 promoter by DNMT-1. Also, confirming a possible therapeutic role, resveratrol was found to partially attenuate TCDD effects [42]. 

Nuclear factor erythroid 2-related factor 2 (NRF2) is also a tumor suppressor that is epigenetically regulated by resveratrol. In a study that looked at the effects of resveratrol on E2 (17β-estradiol)-induced carcinogenesis [43], resveratrol alone up-regulated NRF2 in mammary tissues of rats, and attenuated the repressive effects of E2 on NRF2. E2 suppressed NRF2 through DNA methylation, an activity that was inhibited by resveratrol, thus providing further evidence in support of its regulation of promoter DNA methylation in breast cancer.

#### 3.1.3. DNMTs as Mediators of Resveratrol Effects in Breast Cancer

There is much evidence supporting an inhibitory effect of resveratrol against DNMTs [40,41,44,45,46]. However, most of the evidence comes from in vitro studies. To validate these findings, a pilot study was conducted that enrolled 39 women with increased breast cancer risk [47]. The subjects were divided into three groups—placebo and those receiving 5 or 50 mg resveratrol. Resveratrol was administered twice daily for twelve weeks and the focus was on the DNA methylation of four cancer-related genes—p16, Ras Associated Domain family-1α (RASSF-1α), Adenomatous Polyposis Coli (APC) and Cyclin D2 (CCND2). An inverse relationship between serum resveratrol levels and RASSF-1α methylation was observed, i.e., when resveratrol levels increased, the methylation of RASSF-1α decreased, thus leading to the expression of this tumor suppressor gene. In another study by this group [48], an in vivo effect of resveratrol on DNMT expression was examined in normal vs. tumor tissues. The model evaluated was ACI rats, an inbred line derived from the August and Copenhagen strains. An interesting observation was that resveratrol affected DNMT3b, but not DNMT1. Two different doses of resveratrol were tested and while DNMT3b differed in normal vs. tumor tissues of rats treated with low resveratrol, a high resveratrol dose resulted in decreased DNMT3b in tumor tissues with increased DNMT3b in the normal tissues [48]. This observation is a little different from the one performed in vitro in immortalized breast cancer epithelial cells, MCF10A [49], where resveratrol, at a non-cytotoxic dose, only induced subtle changes in the DNA methylation of eight pre-determined genes. However, the involvement of DNMT3b in resveratrol activity was also identified in a genome-wide DNA methylation study in breast cancer cells [37].

Further confirming the importance of targeting DNMTs in breast cancer patients, the elevated expression of DNMT transcripts was reported in a study that evaluated breast cancer tissues from 40 breast cancer patients and compared those with tissues from 10 paired normal breast tissues [50]. This study further confirmed the down-regulation of DNMT transcripts in vitro in breast cancer cells.

### 3.2. Glioma

Glioblastoma multiforme was the other malignancy where evidence of the effect of resveratrol on methylation was initially presented. It was shown that resveratrol could sensitize resistant glioblastoma T98G cells to temozolomide, inhibiting temozolomide IC-50 with increased apoptosis [51]. Interestingly, temozolomide-resistant cells have increased MGMT (O(6)-methylguanine-DNA-methyltransferase) activity and the protein expression of MGMT is an important determinant of temozolomide-resistance [51]. These results are suggestive of yet another DNA methylation-suppressing activity of resveratrol. In a recent report, inhibition of Wnt signaling has been identified as the mechanism by which resveratrol induces cell death in T98G cells [52]. Epigenetic regulation has been identified as one of the bases of perturbed Wnt signaling [53] and it is plausible that Wnt signaling might be another piece in the puzzle.

### 3.3. Lung Cancer

Lung cancer, the majority of which is non-small cell lung cancer (NSCLC), is the leading cause of cancer-related deaths in the United States, as well as worldwide. A number of studies have described the inhibitory effects of resveratrol against various lung cancer models [54,55,56], albeit, a majority of the studies have focused on NSCLC with little information on the other lung cancer types. In recent years, efforts have been made towards the personalized management of lung cancer [57], with a focus on epigenetics in such personalized therapy [58]. Further, it has recently been demonstrated that resveratrol can epigenetically regulate the expression of zinc finger protein36 (ZFP36) through differential DNA methylation [59]. Specifically, resveratrol reduced the methylation of ZFP36, resulting in its up-regulation in A549 NSCLC cells. A role of ZFP36 in human malignancies is increasingly being realized, making it an attractive target for therapy [60]. Thus, its epigenetic targeting by resveratrol further underlines the anticancer potential of resveratrol, particularly in lung cancer. 

## 4. Acetylation

Even before the realization of the methylation potential of resveratrol, it has been known to modulate acetylation within the cellular microenvironment. A quarter century back, resveratrol was reported to be an activator of sirtuin-1 (SIRT1), the NAD-dependent deacetylase, marking its influence on acetylation, and thereby epigenetic regulation [61]. While the activity of resveratrol against Class III HDACs sirtuins is well characterized, it has been suggested that perhaps resveratrol possesses a pan-HDAC inhibitory property and can inhibit HDACs representing class I, class II as well as class IV [62]. Recently, resveratrol has been identified as an inhibitor of bromodomains [63] and the bromodomain and extraterminal (BET) family [64]. Bromodomains affect epigenetic machinery by recognizing lysine acetylation on histones, thereby functioning as epigenetic readers. The interactions of resveratrol with bromodomains open yet another mechanism of epigenetic regulation by resveratrol which is not yet fully explored. The next few subsections discuss the reported effects of resveratrol on the acetylation of different genes in various human cancers.

### 4.1. Breast Cancer

**Figure 3:**
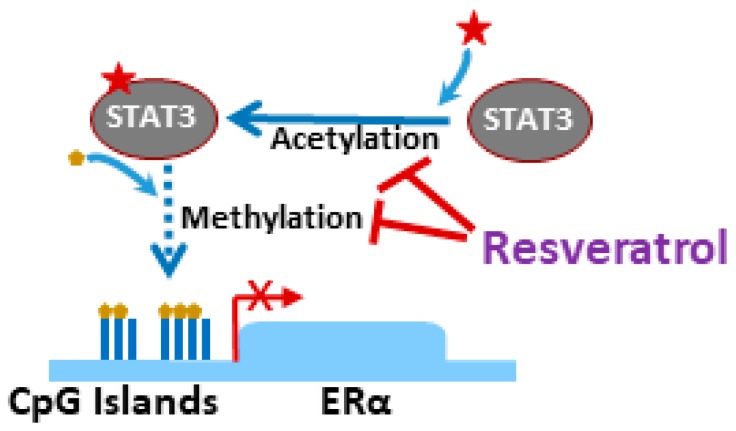
Epigenetic regulation in triple negative breast cancers (TNBCs). TNBCs are characterized by activated STAT3 signaling, involving acetylated STAT3. ERα signaling in TNBCs is silenced through promoter DNA hypermethylation which might be related to STAT3 acetylation but the mechanisms remain unclear (and are therefore shown with a dotted line). Resveratrol is an effective inhibitor of STAT3 acetylation as well as ERα promoter DNA methylation. Restoration of ER-signaling makes TNBC cells sensitive to the ER-targeting therapy, tamoxifen.

Acetylation and its impact on breast cancer progression is well appreciated. It is because of this knowledge that HDAC inhibitors still remain an attractive therapeutic strategy against breast cancers [65,66,67]. It is not only the HDAC inhibitors but also the inhibitors of HATs (histone acetyltransferases) that are being evaluated [68], which provides a good example of how dynamic the process of acetylation and deacetylation is, and how an imbalance can lead to tumor onset and progression. In a study [69] that is indicative of an intricate connection between methylation and acetylation, the two classical epigenetic modifications, it was reported that lysine acetylation within the signal transducer and activator of transcription 3 (STAT3) can impact the interaction of DNMT1 with STAT3 and is accompanied by the demethylation and, thereby, the re-expression of tumor suppressor genes. This study used resveratrol as an acetylation inhibitor and the observations in triple negative breast cancer (TNBC) were further confirmed in melanoma. TNBCs are characterized by the absence of the estrogen receptor (ER) and progesterone receptor (PR), as well as human epidermal growth factor receptor 2 (HER2), and it has been reported that the absence of the *ER*α gene in tumor cells is often a result of methylation [70]. With the observation that STAT3 is acetylated and, therefore, highly expressed in TNBCs, it was evaluated whether inhibiting STAT3 acetylation could reactivate *ER*α [69]. The TNBC cell line MDA-MB-468 was used as the model and it was observed that treatment with resveratrol significantly reduced STAT3 acetylation as well as *ER*α gene promoter DNA methylation (Figure 3). This resulted in the increased expression of *ER*α and the sensitization of otherwise resistant cells to the ER-targeted therapy, tamoxifen. Further, growth of in vivo tumors in mice was not significantly reduced by tamoxifen alone, as expected, but by the combinational treatment comprising resveratrol and tamoxifen, thus validating the in vitro findings [69]. As further evidence of the effect of resveratrol on acetylation in breast cancer, in MCF-7 breast cancer cells, resveratrol has been shown to induce H3 acetylation [71]. Thus, there seems to be evidence suggesting a modulatory effect of resveratrol on protein as well as histone acetylation.

### 4.2. Cervical Cancer

In cervical cancer models, paired box gene1 (PAX1) is a tumor suppressor whose expression is repressed during tumorigenesis by DNA hypermethylation. In a study that evaluated the effect of nutraceuticals, including resveratrol, on the inhibition of cervical cancer through the reactivation of PAX1, it was reported that resveratrol was capable of reactivating PAX1 expression in Caski cells [72]. However, surprisingly, the reactivation of PAX1 was not found to be due to the effect of resveratrol on the DNA methylation of PAX1 promoter. Rather, it possibly involved the acetylation modulating ability of resveratrol through its regulation of HDAC activity. Similar to an effect of resveratrol on histone H3 acetylation in breast cancer cells above, resveratrol has been reported to induce H3 acetylation in HeLa cervical cancer cells as well [71]. Such an effect of resveratrol on H3 acetylation assumes significance given the proposed role of histone H3 acetylation as a prognostic marker for cervical cancer patients [73]. 

### 4.3. Colon Cancer

NF-κB signaling is known to be important to the progression of colon cancer [74], especially in resistance to cisplatin [75]. An increase in the protein acetylation of the NF-κB p65 subunit leads to the activation of the NF-κB pathway and its nuclear accumulation. Therefore, the inhibition of the acetylation of p65 can potentially be an effective strategy to check the growth of colon cancer as well as to overcome resistance to cisplatin. In an in vitro study performed in HK2 cells, resveratrol was found to decrease the protein acetylation of the p65 subunit, thus reversing the cell viability-inducing activity of cisplatin [76]. Such down-regulation of NF-κB protein acetylation by resveratrol in colorectal cells was confirmed in another study [77] and this resulted in reduced tumor invasion and metastasis because of the down regulation of NF-κB-regulated factors, such as MMP-9 and CXCR4.

### 4.4. Leukemia and Lymphoma

In leukemia, resveratrol can potentiate the activity of HDAC inhibitors [78], while in a Hodgkin lymphoma represented by L-428 cells, resveratrol can effectively induce apoptosis as well as cell cycle arrest [79]. As the mechanism, it was observed that resveratrol induced the tumor suppressor p53 through an increase in the p53 K373-acetylation (Table 1). Additionally, resveratrol treatment was found to induce the lysine acetylation of FOXO3a [79]. In leukemia U937 cells, resveratrol potentiates reactive oxygen species production by retinoic acid, particularly the production of superoxide anions, primarily through the up-regulation of the gp91-phox gene that is part of the membrane-bound cytochrome b_558_ [80]. As a mechanism, it was elucidated that resveratrol promoted acetylation within the promoter region of the gp91-phox gene, particularly the Lys-9 residues and Lys-14 residues of histone H3 within the chromatin surrounding the gene promoter.

### 4.5. Prostate Cancer

In prostate cancer, metastasis-associated protein 1 (MTA1) is oncogenic with its expression correlating with tumor progression. It is itself involved in the transcriptional repression of target genes through the post-translational modifications of histones as well as non-histones by virtue of it being a part of the nucleosome remodeling deacetylation corepressor complex, the ‘NuRD complex’. Resveratrol was shown to down-regulate MTA1, leading to acetylation and the activation of the tumor suppressor p53 through the destabilization of the corepressor complex [81]. The NuRD complex plays a role in maintaining chromatin conformation, which it achieves through the deacetylation of histone proteins [82]. HDAC1 and HDA2 are components of the NuRD complex, with the MTA1-HDAC1 subunit responsible for the deacetylation of histones by NuRD. With a direct regulation of MTA1, and the observation that HDAC1 was decreased in resveratrol-treated MTA1 immunoprecipitates, it is evident that resveratrol has a profound effect on histone acetylation. Further, the effects of resveratrol were similar to those of the HDAC inhibitor SAHA, thus underlying the acetylation-affecting epigenetic activity of resveratrol. The results were further confirmed in vivo in a follow-up study [83], and it was shown that the MTA1-mediated tumor progression was, in part, due to PTEN inactivation and that resveratrol could acetylate and reactivate PTEN [84] (Table 1).

Signaling through the androgen receptor (AR) is important in prostate cancer, even in the advanced castrate-resistant prostate cancers. In a study that specifically looked at the regulation of AR signaling by resveratrol, it was observed that treatment with 10 μM resveratrol for just 3 h inhibited the acetylation of AR and affected the binding of AR to the enhancer region of prostate-specific antigen (PSA) [85]. At a slightly longer treatment of 24 h, resveratrol inhibited the nuclear accumulation of AR as well. Given the important role that AR plays in prostate cancer, such epigenetic regulation of its activity and the effect on down-stream signaling by resveratrol is an encouraging finding that gives hope for its possible use as a therapy against prostate cancer.

## 5. Conclusions and Perspectives

While various cellular signaling pathways and genes (oncogenes as well as tumor suppressor genes) are still being evaluated as therapeutic targets of anticancer agents such as resveratrol, in recent years, attention has also turned to epigenetic regulation. In fact, epigenetic regulation of classical signaling pathways is increasingly being realized. For example, two of the very well characterized signaling pathways, NF-κB and STAT3, are epigenetically regulated by resveratrol [69,76,77,86]. This represents a fundamental evolution in our understanding with regards to the intricate regulation of oncogenic pathways. Our discussion on the topic, as presented in this article, detailed many studies that provided evidence supporting the epigenetic activity of resveratrol. However, resveratrol does not regulate gene expression only through epigenetic mechanisms. For example, in a study in acute lymphoblastic leukemia [87] that looked at the possible effect of resveratrol on the methylation of MDR1 (multidrug resistance gene 1), no evidence of the differential DNA methylation of MDR1 promoter by resveratrol was found. While resveratrol had a visible suppressive effect on MDR1, there did not seem to be any epigenetic perspective. This is a perfect reminder that not all regulation of gene expression and function by resveratrol has an epigenetic basis. Additionally, regulation through microRNAs (miRNAs) is within the realm of epigenetic regulation, but we decided to cover just the classical epigenetic mechanisms with regards to methylation and acetylation so as to keep the discussion more focused. Finally, the bioavailability of resveratrol still remains a concern, but that should not deter us from fully elucidating its mechanism of action and its potential as a viable anticancer lead. Several groups are working hard on improving the bioavailability through novel approaches and once they achieve success, resveratrol should be ready for further evaluations in clinical settings as an anticancer agent with multifaceted epigenetic activity.

## Figures and Tables

**Table 1:** Epigenetic effects of resveratrol on tumor suppressors: mechanisms of their re-activation.

Tumor Suppressor	Cancer Type	Effect of Resveratrol	Reference
BRCA-1	Breast	Reduced promotor DNA methylation in vitro	[36]
		Reduced promotor DNA methylation in vivo	[37]
NRF2	Breast	Reduced promotor DNA methylation	[38]
p53	Lymphoma	Induced acetylation	[73]
	Prostate		[75]
PAX1	Cervical	Regulation of histone acetylation	[66]
PTEN	Breast	Reduced promoter DNA methylation	[35]
	Prostate	Acetylation and activation	[77]
RASSF-1α	Breast	Reduced DNA methylation	[42]
